# Using the node importance of patent network to evaluate patent relational value

**DOI:** 10.1371/journal.pone.0325998

**Published:** 2025-07-01

**Authors:** Jianming Guo

**Affiliations:** 1 School of Information Management, Nanjing University, Nanjing, Jiangsu, China; 2 Jiangsu International Joint Informatics Laboratory, Nanjing University, Nanjing, Jiangsu, China; Asansol Polytechnic, INDIA

## Abstract

Citation serves as a common and considerable metric for evaluating the relational value between patents and technologies. This relational value, generally, can be measured by the centrality of the patent citation network. Some centrality indicators can indicate the importance of patents in the citation network, but they ignore the structural information of neighborhood patents. The structural importance of patent network is defined and calculated by considering the degree of similarity between patents and their neighboring node pairs. Briefly, we pair the “neighbor patent” of the target patent and the “neighbor patent” of these “neighbor patents”, called “node pair”. On this basis, we measure the relational value of the target patents. The structure analysis method of patent citation network improves patent value evaluation method from a network science perspective. Firstly, a comprehensive patent citation network is constructed. Secondly, the degree of similarity of patents and their node pairs is used to characterize their local network structural importance, and based on this, PNII, a patent node importance index, is proposed for patent value evaluation. Finally, we applied SIR model to calculate the actual propagation influence of patents, which is used as a criterion to compare the evaluation effect of PNII and other centralities. The patent relational value evaluation result shows that the PNII based on the node importance of patent network is more scientific and accurate than the general network centralities.

## 1. Introduction

High value patents hold significant potential value for innovation subjects; therefore, it is imperative for them to effectively evaluate the worth of their patents to ensure proper structuring. The value of patents is not fixed but dynamic. As new technologies emerge, they have the capacity to replace older technologies, rendering them partially or potentially entirely obsolete and consequently less valuable. In addition, patent value can be roughly classified into individual value and relational value, according to different perspectives or methods. Patent attribute indicators, such as patent family size and pure citation frequency, can effectively reflect the individual value of patents, while patent network analysis can measure the relational value between technologies. Patent citation network records and preserves important information in the process of technology evolution and transmission. This means that we can use citation network to evaluate patent value in the context of technology innovation.

Various motivations exist for studying the value of patents, such as identifying important patents. The evolution process of technology is recorded by the patent citation network, especially some key patents with high technical value may occupy a hub in the network. Using relevant methods can help us measure the importance of nodes in patent citation network, so we can measure the importance of patents and evaluate their relational value.

The citation relationship between patents reflects the flow of technical knowledge and determines a patent’s position in the evolving network of technical knowledge. This positioning allows a patent to exert influence and ultimately reflects its value. Among the indicators of value, patent citations are highly regarded and extensively researched. It can be concluded that patents with more frequent citations tend to generate higher technological value.

From the citation relationship, patent co-citation and bibliographic coupling relationship can be extended. The dimensions of different relationships reflecting the value of patents may be different, for example, patents with co-citation relationship usually have similar knowledge structure, while bibliographic coupling relationship can reveal the thematic relevance of sub-technical fields through cluster analysis. All these citation-based analyses provide valuable information that should be integrated rather than treated in isolation. Over the past two decades, researchers have developed and applied methods that combine relationships based on multiple citations to predict research frontiers [1–2]. However, less research has been conducted to assess the value of patents using combinations of multiple citation relationships. Some scholars have proposed a method called “Comprehensive Patent Citation Network (CPC)” to effectively combine multiple citations [3].

The essence of patent relational value evaluation based on the patent network is essentially a problem of ranking nodes in network. Centrality is a measure of a node’s ability to acquire and control resources, and nodes with higher centrality have relatively higher importance in the network [4]. Different centrality measures reflect different aspects of patent value. Degree centrality measures the extent to which a node is directly connected to other nodes in the network, reflecting its local importance. Patents with high closeness centrality have a greater ability to influence subsequent patents and are therefore more valuable [5]. And high betweenness centrality patents can be considered as hubs in technological communication and dissemination [6]. Moreover, patent networks mostly belong to complex networks [7], and methods for evaluating identified key nodes in complex networks [8] may provide insights for the comparation of different evaluation methods of patent relational value.

Drawing on network science methods and technical knowledge diffusion theory, we propose a method to assess patent relational value based on the node importance of patent citation network. The first step constructs a comprehensive citation network for patents. The second defines and calculates the patent network importance index (PNII). The final step involves measurement validation, which applies SIR model to compare different evaluate methods of patent relational value.

## 2 Literature review

### 2.1 Patent citation network

Patent documents, as an important manifestation of technological innovation, have great economic and technological value. Through the analysis and excavation of patent documents, we can deeply portray the development of technical fields, analyze the path of technological evolution, and excavate important patents and key technologies. Yoon et al. [9] first put forward patent network analysis method, and the network constructed by integrating a variety of citation relationships can be called the comprehensive patent citation network [3]. From the perspective of technology life cycle, there are certain key moments in the process of technology development and evolution, such as the generation of key core technologies, etc., and this important information is recorded and preserved by the patent citation network.

As the examination of citation relationships has intensified, there has been a gradual expansion in the types of citation-based relationships studied. Bibliographic coupling [10] and co-citation [11], by citing the same patent or being co-cited by other patents, indicate a sharing relationship between two patents. There is no time limit on the sharing relationship, and at the same time, since the sharing relationship reflects the network structural characteristics, it can reveal implicit knowledge flows that are not available from direct patent citations.

The concept of indirect citation, previously referred to as ‘vertical coupling’, and its related research have a historical background extending over several decades [12]. It is reasonably foreseeable that indirect citation arises from direct citation and implicitly constitutes a vein of technological evolution and technological knowledge flow. The connection to the underlying patent is unveiled through indirect links, which are captured within the citation chain [13].

Analysis of the four networks above provides a comprehensive picture of patent network structure and function [12,14]. Approach based on single relationship may result in an incomplete comprehension of knowledge flow dynamics and hinder the objective assessment of patents’ value [3]. Hence, it is necessary to consider the impact of multiple relationships on patent value assessment in a comprehensive manner.

A patent-comprehensive citation network refers to a network formed by combining multiple citation relationships. Analysis based on comprehensive patent citation networks is becoming popular. A study conducted by Yang et al. [3] evaluated patent value based on a comprehensive citation network, yielding more accurate results than direct citation network. We will follow Yang’s method to construct a comprehensive patent citation network in this study.

### 2.2. Centrality and patent relational value

Centrality refers to a node’s measure of accessing and controlling resources. A node with high network centrality indicates direct or indirect connections with a greater number of others, leading to them a greater influential [15]. Therefore, nodes with higher centrality hold a higher relative importance in the network, enabling them to exert more power over other nodes and avoid restrictions from them. The centrality of the network based on patents relationship reflects patent relational value. Among many centralities, degree centrality, closeness centrality and betweenness centrality are the most classic and widely used.

Patents with higher degree centrality have more connections, informal rights, and influence. They fulfill the three essential requirements of a patent—utility, novelty, and inventiveness [5,16]. Thus, citations form the technical foundation of a patent.

Closeness centrality measures the combined shortest distances between a node and all other nodes in the network. It reflects an actor’s ability to avoid control by others while emphasizing efficiency and independence [17]. Efficiency indicates the ability to reach other actors in the fewest steps possible, while independence implies relying less on intermediaries to establish contact. The greater the closeness centrality, the greater the ability to influence subsequent patents, making them more valuable. Patents with higher closeness centrality have greater relational value because they have a more ability to influence subsequent patents.

Comet [18] argues that a node positioned in a structural hole facilitates the transfer of resources or information between distinct clusters or nodes, thus increasing the potential for value creation. Within the patent citation network, intermediaries play a crucial role in transferring technical knowledge across different fields of expertise. By establishing connections between patents in diverse domains, they enhance the likelihood of generating novel technologies and fostering technological advancements. The higher the betweenness centrality of patent, the greater its value, because its role as the intermediary of technology development is more significant [5–6].

The essence of using patent networks to assess the value of patents is to rank their importance. Research has shown that point centrality is a simple and effective local algorithm, and the feature factor, PageRank, HITs centrality and H-index can compensate to a certain extent for the limitation of considering only the number of citations but not the quality of citations; the calculation of closeness centrality and intermediary centrality requires global information of the network, and the complexity of the algorithm is high, which has limitations in application. For this reason, some scholars calculate node importance based on the characteristics of node local network topology, and then effectively balance the relationship between computational accuracy and algorithm complexity. Ruan et al [8] proposed a node importance evaluation algorithm that integrates the degree and neighbor node topological overlap by quantifying the similarity between nodes defined by node local network topology, which in turn effectively balances the relationship between computational accuracy and algorithm complexity.

Through the approaches mentioned above, several unresolved issues remain, which can be summarized in two points. Firstly, how can the importance of patents in the network be more comprehensively characterized? Chen et al. [19] argue that patent citation analysis may be inaccurate due to different relationships. Atallah and Rodríguez [20] suggest that not all citations hold equal value, and citations from varying quality ought to be assigned distinct ratings. They recommend combining various patent citation metrics to estimate patent value. Therefore, it is necessary to select or develop an appropriate metric within the patent network to effectively characterize patents’ value. The common centralities neglect the structure information of the neighboring patent when evaluating the value of the patent relationship. We will therefore follow Ruan’s approach to define and calculate the structural importance by considering the similarity between patents and their neighboring node pairs.

Assessing patent relational value from a citation network perspective poses another challenge due to the absence of readily available criteria for verifying the reliability of patent valuation results. Researchers have developed various indirect indicators, such as patent family and maintenance [3,21]. However, these indicators remain at the patent level and may not necessarily apply to the outcomes of patent network analysis. Therefore, an attempt is made to validate the results of patent value assessment by employing the node importance evaluation method in complex networks. The commonly used methods include those based on network propagation dynamics [22] and those based on network robustness and vulnerability [23]. In this study, we will select the network-based diffusion dynamics model approach, considering that patent networks, as a comprehensive repository of technological knowledge, can depict the diffusion process of technological knowledge.

## 3. Methods

By constructing a comprehensive patent citation network, we leverage the similarity between neighboring node pairs within the network to characterize their local structure importance. This serves as the foundation for establishing a patent node importance index (PNII). Utilizing this index, we can assess the value of patent relationships within a specific technological domain. To evaluate the outcomes, we rely on the SIR (Susceptible, Infected, Recovered) model for analysis.

### 3.1. Construction of comprehensive patent citation network

Patent citation network is constructed with patents as nodes and citation relationships as edges. There are mainly four kinds of citation relationships between patents [3], as shown in **Table 1**.

**Table 1:** Four citation relationships between patents.

Features	Direct Citation	Indirect Citation	Co-citation	Bibliographic Coupling
Abbreviation	DC	IDC	COC	BC
Relationship types	Citation	Citation	Co-occurrence	Co-occurrence
Direction	Directed	Directed	Undirected	Undirected
Weight	Binary	Multi-valued	Multi-valued	Multi-valued

The types of patent citation relationships are divided into real relationship (direct citation) and artificial relationships (indirect citation, co-citation, and bibliographic coupling) [24]. Real relationship can directly indicate the process of knowledge diffusion among patents, while artificial relationships can indirectly indicate the process by calculating the strength of the relationship. The patent network based on single relationship is limited in its sparsity to accurately depict the flow of technology [12]. By integrating both real and artificial relationships, constructing a comprehensive patent citation network offers a more detailed view of the interconnections within patent technologies [13]. This approach not only enhances the evaluation models for patent quality [20] but also facilitates a more precise assessment of patent value [3].

Generally, the network is represented by a graph G=(V,E), where V denotes the set of vertices and E denotes the set of edges. For computational convenience, graphs are often represented by adjacency matrices, and for G with n patent nodes can be represented by a n-row and n-column adjacency matrix A as:


$$A_{ij}=\left\{ \begin{array}{c} 1ifpatentjcitedpatenti\\ 0otherwise \end{array}\right.$$ (1)

In this way, the patent direct citation network can be expressed as DC=A, and the other three citation relationship networks can be obtained by A operation, which is calculated as shown in equations (2)–(4).


$$IDC=A^{N+1}$$ (2)

$$COC={A\times A}^{T}$$ (3)

$$BC=A^{T}\times A$$ (4)

IDC denotes patent indirect citation network. $A^{N+1}$ denotes the matrix A does (N+1) times multiplication operation to get patent Nth order indirect citation network. N=1 means only first-order indirect citation relationship is considered in this paper. If there exist nodes i,j,k, node i cites node k and node k cites node j, then $A_{jk}\cdot A_{ki}=1$, which means there is an indirect citation relationship between node i and j.

COC denotes the patent co-citation network. The co-citation relationship indicates that two patents are cited by other patents at same time, $A^{T}$ denotes the transpose matrix of A, and COC is a symmetric matrix. If there exist nodes i,j,k, and node k cites nodes i and j, then $A_{ik}\cdot A_{jk}=1$, which means that there is a co-citation relationship between node i and j.

BC denotes the patent coupling network. The coupling relationship indicates that two patents cite other patents at the same time, $A^{T}$ denotes the transpose matrix of A, and BC is also a symmetric matrix. If there exist nodes i,j,k and both node i and node j refer to node *k*, then $A_{ki}\cdot A_{kj}=1$, which indicates that there is a coupling relationship between nodes i and j.

In addition to direct citation, indirect citation, co-citation and coupling all have strength of relationship. The higher the relationship strength, the more obvious the “knowledge flow” between patent pairs is, and the higher the reflected technical value is. To construct a comprehensive patent citation network, a relationship strength threshold needs to be set in advance before extracting high-strength relationships [3]. Three relationship strength matrices are defined as idc, coc and bc, and the number of indirect citations is used to represent the indirect citation relationship strength, i.e.,idc=IDC; the co-citation and coupling relationship strength matrices are calculated as shown in equations (5)–(6).


$$\begin{array}{c} {coc}_{ij}=\sum {\mathrm{\backslash nolimits}}_{k=1}^{n}\;\frac{A_{jk}\cdot A_{ik}}{m_{k}-1} \end{array}$$ (5)

where $A_{jk}\cdot A_{ik}=1$ when nodes i and j are cited by node k at the same time, and 0 otherwise, $m_{k}$ denotes the number of patents cited by node k, and n denotes the total number of patent nodes.


$$\begin{array}{c} {bc}_{ij}=\sum {\mathrm{\backslash nolimits}}_{k=1}^{n}\;\frac{A_{kj}\cdot A_{ki}}{n_{k}-1} \end{array}$$ (6)

where $A_{kj}\cdot A_{ki}=1$ when nodes i and j refer to node k at the same time, and 0 otherwise. $n_{k}$ denotes the number of node k being referenced and n denotes the total number of patent nodes.

It is necessary to set certain thresholds for screening the strength of the relationship. The three relationship strength thresholds are η, λ and γ respectively, and the upper quartile of the matrix data is taken as the strength threshold, i.e., the top 25% of the data of each type of network is selected as the screening criterion of association strength. This criterion indirectly indicates the knowledge diffusion process among patents by using high-intensity artificial relationship, in line with the idea of “28-law”. According to the equations (7) to (9), we can calculate the citation relationship matrix whose relationship strength is higher than the threshold value, and then calculate the adjacency matrix CPC according to the equation (10).


$$\begin{array}{c} {IDC}_{ij}^{\prime }=\left\{ \begin{array}{c} 1{idc}_{ij}>\eta \\ 0\text{ otherwise } \end{array}\right. \end{array}$$ (7)

$${COC}_{ij}^{\prime }=\left\{ \begin{array}{c} 1{coc}_{ij}>\lambda \\ 0\text{ otherwise } \end{array}\right.$$ (8)

$${BC}_{ij}^{\prime }=\left\{ \begin{array}{c} 1bc_{ij}>\gamma \\ 0\text{ otherwise } \end{array}\right.$$ (9)

$${CPC}_{ij}=\left\{ \begin{array}{c} 1{DC}_{ij}+{IDC}_{ij}^{\prime }+{COC}_{ij}^{\prime }+{BC}_{ij}^{\prime }\geq 1\\ 0\text{ otherwise } \end{array}\right.$$ (10)

Since the diagonal element ${CPC}_{ii}=0$ of the adjacency matrix, the network can be abstractly represented as a directed unweighted simple graph, and then the relevant theoretical knowledge can be used to measure node importance.

### 3.2. Importance of network nodes

The importance of a node in a network depends not only on the degree of the node itself, but also on the dependence of the neighboring nodes on that node, where the neighboring nodes specifically refer to the low-order neighboring nodes within two hops [8]. Each patent in the patent network can be regarded as a technological whole composed of multiple knowledge, and the citation relationship between patents is the process of knowledge spillover and flow, which some scholars call technology diffusion [25]. The phenomenon of technology diffusion reflects the law of technology innovation, and reflect the phenomenon of technology diffusion more objectively, which can reveal the process of spreading, promoting and applying new technologies among potential users or inventors [26]. For a patent network, a patent represents a node, and the reference of a patent by subsequent patents will lead to the continuous expansion of technical knowledge diffusion, which will lead to the evolution of the whole network. Subsequent patents will be laid out to form neighboring nodes around the initial node. From the patent perspective, the neighboring patent nodes within its two hops are the patents with direct citation and one indirect citation relationship, which are called neighboring patents in this paper.

**Fig 1:**
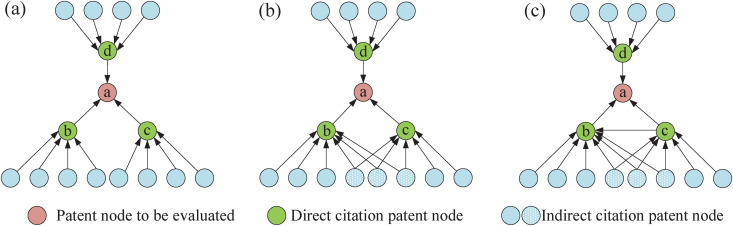
Schematic calculation of similarity between neighboring patents. (a). The neighbors of the neighbor patents do not overlap. (b). The neighbors of the neighbor patents overlap. (c). Neighbors of neighbor patents overlap and there exist citation between neighbor patents.

In patent citation network, a patent node is usually surrounded by many neighboring patents. The importance of the patent node can be quantitatively characterized by simply comparing the similarity between neighboring nodes two by two. As shown in **Fig 1**, the circles indicate the nodes in the patent network, and the arrows indicate the citation relationship, i.e., patents *b*, *c* and *d* cite patent *a*, while other patents indirectly cite patent *a* by citing patents *b*, *c* and *d*. Although the indegree of patent node *a* is smaller than its neighboring patent nodes *b*, *c* and *d*, from the perspective of network structure, when patent node *a* is removed, the patent network will be split into three subnets. This seriously disrupts the network structure because some technology relationships are lost (e.g., the coupling relationship between patent *b* and *c*). From the perspective of information dissemination, additionally, all other nodes cite patent *a* directly or indirectly, indicating that patent *a* is the source of technical information. The technical information of patent *a* will spread to a wider technical field. As depicted in **Fig 1(b)**, if patents *b* and *c* are both cited by the same set of patents, they share a common citation from three different patents. This indicates a co-citation relationship between patents *b* and *c* with a strength of 3, reflecting their interconnectedness. At this time, even if patent *a* is deleted, most of the nodes in the patent network are still connected, so the close technical relationship between neighboring patents will weaken the importance of patent node *a*. **Fig 1(c)** adds the citation relationship of patent *c* to patent *b* based on **Fig 1(b)**. At this time, even if patent *a* is deleted, patent *b* will become a new source of technical information, further weakening the importance of patent *a*.

The analysis demonstrates that the lower the degree of similarity between neighboring node pairs of patents, the more the patent network relies on that node, thereby indicating a higher relational value for that node. Therefore, the difference degree between neighboring node pairs can be used to characterize the importance of patents, and then define the patent node importance index (PNII), which is calculated as shown in equation (11).


$$PNII(i)=\left\{ \begin{array}{c} 0,0\leq k_{i}\leq 1;\\ \sum {\mathrm{\backslash nolimits}}_{b,c\in n(i)}\;(1-sim(b,c)),k_{i}\geq 2 \end{array}\right.$$ (11)

where$k_{i}$ denotes the number of neighboring nodes of patent node i. When there is no neighboring node or only 1 neighboring node of patent node, its PNII value is 0. When $k_{i}\geq 2$, n(i) denotes the set of neighboring nodes of node i, and sim(b,c) denotes the Jaccard similarity of neighboring nodes to patents *b* and *c*, which is calculated as shown in equation (12).


$$sim(b,c)=\left\{ \begin{array}{c} \frac{\left|n(b)\cap n(c)\right|}{\left|n(b)\cup n(c)\right|},\text{ if there is no reference between b and c}\\ 1,\text{ otherwise } \end{array}\right.$$ (12)

where n(b) and n(c) denote the sets of patent nodes citing patents b and c, respectively, |n(b)∩n(c)| denotes the number of patents in the set n(b)∩n(c), |n(b)∪n(c)| denotes the number of patents in the set n(b)∪n(c), and sim(b,c) denotes the Jaccard similarity of patents b and c, taking values in the range 0–1. A larger similarity indicates a higher degree of overlap between neighboring patent pairs.

Further, when $k_{i}≽2$, Eq. (11) can be deformed into Eq. (13).


$$\begin{array}{c} PNII(i)=C_{k_{i}}^{2}-\sum {\mathrm{\backslash nolimits}}_{b,c\in n(i)}\;sim(b,c) \end{array}$$ (13)

where $C_{k_{i}}^{2}=k_{i}\cdot (k_{i}-1)/2$, denotes the number of neighboring patent pairs of patent i. Equation (13) shows that the *PNII* value of patent *i* is affected by both the number of neighboring patents of patent *i* and the similarity of neighboring patent pairs. The more the number of neighboring patents of a patent or the less the overlap between neighboring patent pairs, the greater the *PNII* value and the greater the importance of the patent.

Take the patent a to be evaluated in **Fig 1** as an example.

In **Fig 1(a)**, PNII(a)=(1−0)+(1−0)+(1−0)=3.

In **Fig 1(b)**, PNII(a)=(1−3/8)+(1−0)+(1−0)=2.625.

In **Fig 1(c)**, PNII(a)=(1−1)+(1−0)+(1−0)=2*.*

Observations reveal that when the number of neighboring patents is constant, a lower degree of overlap among the pairs of patents in the vicinity of patent *a* corresponds to a higher PNII value for patent *a*. This indicates a higher relational value of patent *a* in the network. The calculation results align with our conceptual understanding, thereby confirming the rationality and effectiveness of the PNII.

### 3.3 Evaluation of results

We used the SIR Model to compare the performance of PNII with other centralities in assessing the value of patent relationships. The SIR Model is one of information dissemination model that can simulate the average infection scope for each patent node, thereby reflecting the actual influence of dissemination by these nodes. We employ this influence as a metric for evaluation. The greater the correlation between the simulation outcomes and the centralities assessment, the more effective the index is at evaluation.

In the SIR model, there exist three states of patent nodes:①Susceptible (S);②Infective (I);③Recovered (R). At each time step, the node in the Infective state I will infect the neighboring nodes in the Susceptible state S with propagation probability β, and will enter the state R with recovery probability ε. The nodes in the state R will not be infected again, and there will be no nodes in the Infective state I in the network as time increases.

The influence of a node’s propagation is indicated by its average infection range. In this study, the comprehensive patent citation network is characterized as a directed, unweighted, and simple network, where information dissemination occurs in the reverse direction of the patent citation, flowing from the citing patent to the cited patent.

To calculate the final propagation influence of the node, let the recovery probability ε=1, which means that the node in the infected state at the current time will immediately recover to the state R in the next time step. The information propagation probability threshold is calculated as shown in equation (14).


$$\beta_{0}=\frac{\langle k\rangle }{\left\langle k^{2}\right\rangle }$$ (14)

where ⟨k⟩ is the network average degree and $\left\langle k^{2}\right\rangle$ is the network second-order average degree. Since infected nodes are infected with probability $\beta_{0}$ and the results of each independent experiment may differ, the actual propagation influence Φ(i) of patent node i is defined and calculated as shown in equation (15).


$$\begin{array}{c} \Phi (i)=\frac{1}{L}\sum {\mathrm{\backslash nolimits}}_{l=1}^{L}\;\Phi_{l}^{\prime }(i) \end{array}$$ (15)

where L denotes the number of experimental repetitions, $\Phi_{l}^{\prime }(i)$ denotes the total number of nodes in the network where node i is in the recovery state R as the source of infection in the lth propagation experiment. The average of the repeated experimental results is employed as the actual measure of the node’s propagation influence in equation (15).

Spearman’s rank correlation coefficient was used to measure the accuracy of the ranking results of each centrality index. The correlation between each ranking index and node propagation influence is calculated, and the higher the correlation coefficient ρ is, the closer the ranking method is to the simulation result of SIR model, and the better the result is. The calculation method is shown in equation (16).


$$\rho =\frac{\sum_{i=1}^{n}\;\left(x_{i}-\overset{―}{x}\right)\left(y_{i}-\overset{―}{y}\right)}{\sqrt{\sum_{i=1}^{n}\;{\left(x_{i}-\overset{―}{x}\right)}^{2}\sum_{i=1}^{n}\;{\left(y_{i}-\overset{―}{y}\right)}^{2}}}$$ (16)

where n is the number of patent network nodes, $x_{i}$ and $y_{i}$denote the rank data of the ith patent node of the two indicators corresponding to the converted original data, and $\overset{―}{x}$ and $\overset{―}{y}$ denote the mean value of the rank data of the two indicators.

## 4. Empirical research

We validate the patent relational value evaluation method based on the PNII, as proposed in this study, using specific case studies. The integrated circuit (IC) industry serves as the foundation and driving force behind the rapid development of the information technology industry [27]. The technological level and scale of IC industry development have become crucial for enterprises, economy, and social competitiveness and sustainable development [28–29]. It is both a technology-intensive industry and a capital-intensive industry [30]. ICM technology is divided into eight sub-fields, including cleaning, lithography, etching, thin film, doping, annealing, and planarization, with planarization being an essential step in modern integrated circuit manufacturing and the final stage of chip fabrication process [31].

Planarization technology, a key technique employed in the manufacturing and optimization of microprocessors and other electronic components, plays a crucial role in achieving highly integrated chip designs. By combining multiple layers of electronic devices, planarization enables the realization of compact circuitry with improved performance, density, and power consumption. Evaluating the patent value in this field has become paramount due to its significant impact on enhancing integrated circuitry. Assessing the patent value in the planarization technology domain is not only vital for companies to understand their market competitiveness and technological advantages, but it also supports innovation decision-making and technological collaborations, guiding companies in maintaining their competitive edge and achieving long-term growth. Currently, international competition around planarization technology is primarily concentrated in the United States, Japan, and Germany. This empirical study focuses on patent data in the field of “planarization technology” as an illustrative example to explore this subject further.

### 4.1. Data

The data was sourced from the Derwent Patent Database. The retrieval strategy used was based on Liu et al. (2018) as follows: TI=((Chemical-mechanical-polish* or “Chemical Mechanical Polish*” or Polish* or CMP or SFP or “Stress Free Polish*” or “Stress-free Polish*”) and (IC or Semiconductor or “Integrated Circuit”)). The retrieval was conducted on July 13, 2024. After data cleaning, a total of 11,539 patent records were obtained. The R-Project was utilized to extract patent PN numbers, relevant patent attributes, and patent citation relationships.

The patent citation data downloaded from the Derwent database contains all patents family and their cited patent data. Utilizing a single patent to replace patent family can lead to significant inaccuracies. Therefore, the approach employed in this study for handling patent family is to employ the regular expression “PN–” to extract the citation relationship.

The number of cited patents extracted is 5,127, the number of cited patents is 2,518, and the number of patent citation relationships is 13,185. Given that the cited patents and the citing patents may pertain to different disciplines or technologies, the cited patents might not be included in the initial dataset. Consequently, the direct utilization of citation data could potentially diminish the accuracy of the results. We matched both the citing and cited patents to comprehensively evaluate the patents within the domain. This process resulted in a total of 5,621 patents within the domain and 12,608 patent citation relationships being successfully matched. After the matrix calculation from Eq. (2) to Eq. (10), the constructed comprehensive patent citation network contains a total of 5,621 patents and 33,571 citation relationships.

### 4.2. Evaluation results of patent relational value

The propagation probability threshold of the CPC network is calculated as $\beta_{0}=0.14$. The propagation influence is calculated using the igraph package and self-coded functions in R. We set L to 100, indicating that 100 independent experiments are conducted, and the average result from these experiments is used to determine the actual propagation influence of the patent nodes.

In terms of the overall results, the PNII score reflects the “long-tail distribution” of patent value. To verify the effectiveness of the proposed node importance index PNII, the commonly used network centrality indexes: degree centrality (degree), closeness centrality (close), betweenness centrality (between), and dissemination influence of nodes with propagation probability $\beta_{0}$=0.14 were selected as the comparison indexes. **Table 2** gives the descriptive statistics of the scores of different indicators. It shows that the results of different centrality indicators have different magnitudes, and the distribution of scores also differs. $\beta_{0}$reflects the average influence range of patent nodes in the process of technological knowledge diffusion. Skewness and kurtosis indicators can be used to determine whether the data conform to a normal distribution.

**Table 2:** Descriptive statistics of different centralities.

Index	degree	close	between	PNII	$\begin{array}{c} \mathrm{\backslash bold}\beta_{0} \end{array}$
Min.	1	0.113	0	0.000	1.000
1st Qu.	6	0.196	0	42.140	1.370
Median	10	0.211	949.3	295.360	2.250
Mean	11.94	0.210	24799.2	1502.320	3.519
3rd Qu.	15	0.226	15652.3	1119.710	4.060
Max.	107	0.293	1782116.3	90511.680	47.770
skewness	2.458	−0.372	8.775	8.788	3.650
kurtosis	12.162	0.400	117.483	115.031	20.139

**Fig 2:**
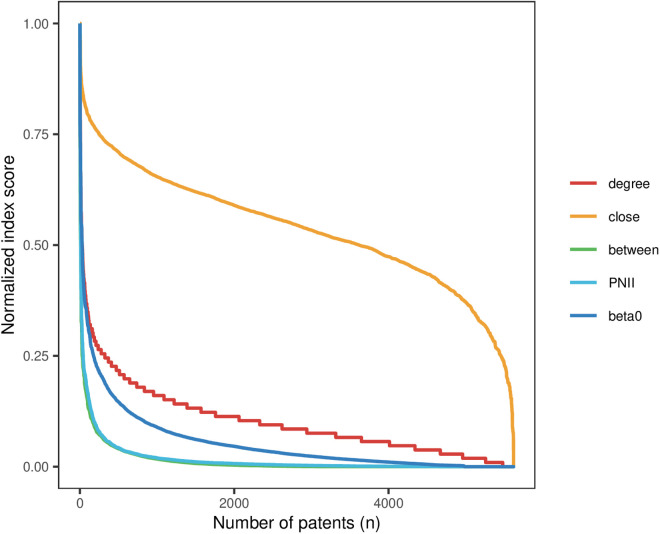
Distribution of scores of different centralities.

Skewness is categorized into three types: normal distribution (with a skewness value of 0), right skewness (with a skewness value greater than 0), and left skewness (with a skewness value less than 0). Similarly, kurtosis is divided into three categories: normal distribution (with a kurtosis value of 0), thick-tailed distribution (with a kurtosis value greater than 0), and thin-tailed distribution (with a kurtosis value less than 0). It is observed that, aside from indicators with skewness and kurtosis values close to those of centrality, the skewness values for the other indicators exceed 3, and the kurtosis values exceed 13. The distribution of scores across different indicators exhibits characteristics of right skewness and thick tails. The scores of various indicators display traits of right-skewed, thick-tailed distributions, suggesting that the patent value follows a “long-tailed” distribution pattern. The distribution of the scores of different indicators is shown in **Fig 2**. The normalized scores are sorted in descending order, revealing that all indicators except for closeness centrality exhibit a “long-tail distribution” in patent value. This observation confirms the validity of the proposed PNII.

### 4.3. Evaluation effect of different centralities

The Spearman correlation coefficient between each ranking indicator and node dissemination influence was calculated. The higher the correlation coefficient ρ, the closer the assessment method is to the simulation results of the SIR model, and the better the evaluation results. The correlation coefficients are shown in **Table 3**.

**Table 3:** Spearman correlation coefficients of different centralities.

Index	degree	close	between	PNII	$\beta_{0}$
degree	1.000	0.682	0.700	0.672	0.472
close		1.000	0.564	0.634	0.378
between			1.000	0.596	0.478
PNII				1.000	0.691
$\beta_{0}$					1.000

At a propagation probability of $\beta_{0}=0.14$, the correlation coefficient between the PNII and the actual propagation influence of the patent reaches its peak at 0.691. This is followed by the correlation coefficients for betweenness centrality and degree centrality, which are 0.478 and 0.472, respectively. In contrast, the correlation coefficient for closeness centrality with the actual propagation influence is the lowest, at 0.378. The correlation results indicate that the PNII is more reasonable and effective.

**Fig 3:**
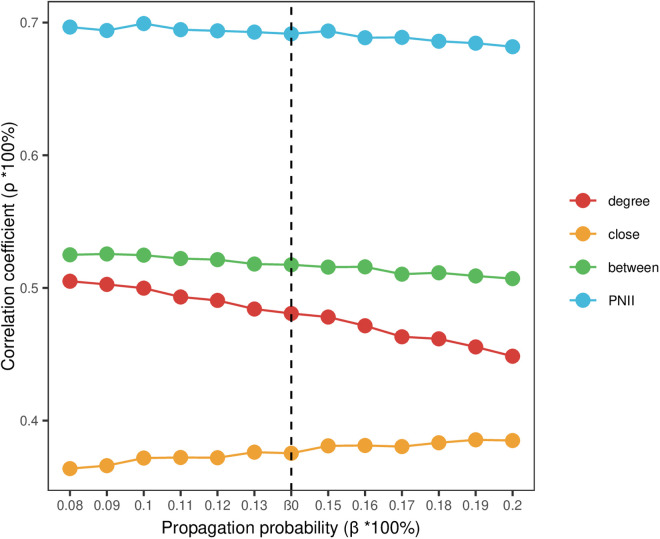
Evaluation effect of each centrality under different propagation probabilities.

The Spearman correlation coefficients of the above four indicators and the communication impact indicators are further compared at different propagation probabilities, and the results are shown in **Fig 3**. The range of different propagation probabilities is set as $\beta =\beta_{0}\pm 0.06$, and the higher the correlation coefficient, the better the indicator identification effect. The findings indicate the following: The correlation coefficients between the PNII evaluation outcomes and the node’s propagation influence are consistently the highest across various propagation probabilities. Following this, the betweenness centrality, degree centrality, and closeness centrality exhibit the next highest correlation coefficients, aligning with the results of the correlation tests. There is a consistent relationship between each indicator and the network’s propagation influence across different propagation probabilities.

**Fig 4:**
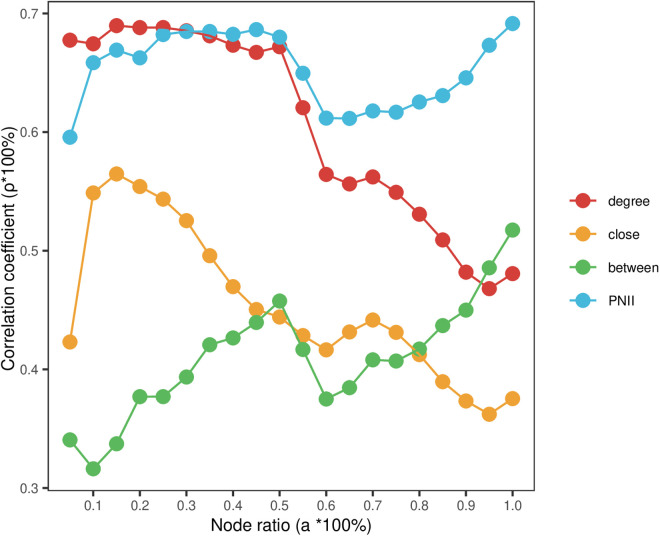
Evaluation effect of each centrality under different node ratios.

The quantity of high-value patents is relatively modest, constituting a minor fraction of the total patent landscape. However, the subject of the aforementioned correlation test encompasses all patents. Consequently, we delve further into the correlation between patent nodes with varying percentages of top rankings, as determined by each indicator, and the actual influence ranking outcomes. The results of the different methods of evaluation are ranked in descending order by their scores and then correlated with the nodes whose node ratio is $a=a_{0}\pm 0.05$. **Fig 4** illustrates that, overall, the PNII evaluation results exhibit the highest level of consistency, with correlation coefficients ranging from 0.6 to 0.7 across various node ratios. The evaluation effectiveness of degree centrality and closeness centrality tends to decrease with different node ratios, whereas betweenness centrality shows an increasing trend. For high-value patents within a narrow range of node ratios, degree centrality outperforms closeness centrality, while betweenness centrality has the poorest recognition capability. When the node ratio falls between 10% and 50%, the evaluation performance of PNII and degree centrality is largely equivalent. Within the top 5% of node ratio, degree centrality yields the most favorable evaluation outcomes. This indicates that the patent citation network embodies the power-law distribution characteristics of complex networks, where a small number of patents have a significantly higher number of connections.

### 4.4. Evaluation effect of different networks

The experiments conducted above have confirmed that PNII demonstrates greater validity and consistency compared to the general centralities. We continue to explore whether CPC have better performance and evaluation results than networks based on single relationships.

**Table 4** displays the basic characteristics of the CPC alongside the four other networks. To assess the disparities among these networks, we have utilized metrics such as the number of nodes, the number of patent pairs, the average degree, and the network density. The number of nodes serves as an indicator of its capacity to evaluate each patent within the network. As illustrated in **Fig 1**, the direct citation relationship (DC) is more general, whereas the relationships IDC, COC, and BC, which are derived from DC, are comparatively stringent. This stringent nature leads to a reduction in the number of patent nodes when analyzing patent information using COC or BC networks. In contrast, the CPC network, which amalgamates various relationships, maintains an unaffected node count.

**Table 4:** Basic characteristics of different networks.

Types of network	Number of connected nodes	Number of isolated nodes	Number of Patent pairs	Degree of average	Network density
CPC	5,621	0	33,571	11.945	0.0011
DC	5,621	0	12,608	4.486	0.0004
IDC	3,925	1,696	15,906	5.659	0.0005
COC	2,065	3,556	17,835	6.346	0.0006
BC	4,560	1,061	64,010	22.775	0.0020

CPC encompasses more patent pairs than all other networks, except for BC. The relationship of CPC is more abundant, which can better show the diversity of the relationship between patents. Similarly, the average degree of the CPC is larger than the other networks except BC, indicating that the average patent is cited 11.945 times, rather than 4.486 times in DC. This shows that CPC helps to supplement the limitations of the single-citation perspective. Network density can represent the number of relationships in the network and the degree of adjacency. The density of all networks is less than 0.002, reflecting the sparse nature of patent networks. However, the density of CPC is significantly higher than that of DC, indicating that the overall structure of CPC is more complete.

Citation time lag reflects the difference of application time between related “patent pairs” and is a key index to evaluate the effect of patent network. The shorter the citation delay is, the better the patent network can evaluate the emerging patents [3]. As can be seen from **Table 5**, the average citation time lag of CPC is 47.73 months, and that of DC is 60.39 months, which reduces the average citation time delay by 12.66 months. This means that CPC are more efficient than DC in evaluating new patent applications or grants after taking other relationships into account.

**Table 5:** Citation lag of distribution of patent pairs.

Types of network	Mean	Median	Standard deviation	Min	Max
CPC	47.73	34	46.89	0	259
DC	60.39	48	46.13	0	259
IDC	95.21	86	52.99	0	253
COC	32.38	21	34.20	0	225
BC	44.99	33	42.35	0	248

Next, we applied PNII to four single-relationship networks to verify whether CPC have better performance and evaluation results. The Spearman correlation coefficient between PNII results and node propagation influence of each network was calculated. The correlation between PNII results of different networks and the propagation influence is shown in **Table 6**. The results show that CPC has the highest correlation with actual propagation influence, followed by DC and COC.

**Table 6:** Spearman correlation coefficients of different networks’ PNII.

Network	DC	IDC	COC	BC	CPC	$\beta_{0}$
DC	1.000	0.694	0.824	−0.234	0.458	0.489
IDC		1.000	0.732	−0.187	0.493	0.396
COC			1.000	−0.201	0.586	0.429
BC				1.000	0.230	0.157
CPC					1.000	0.691
$\beta_{0}$						1.000

**Fig 5:**
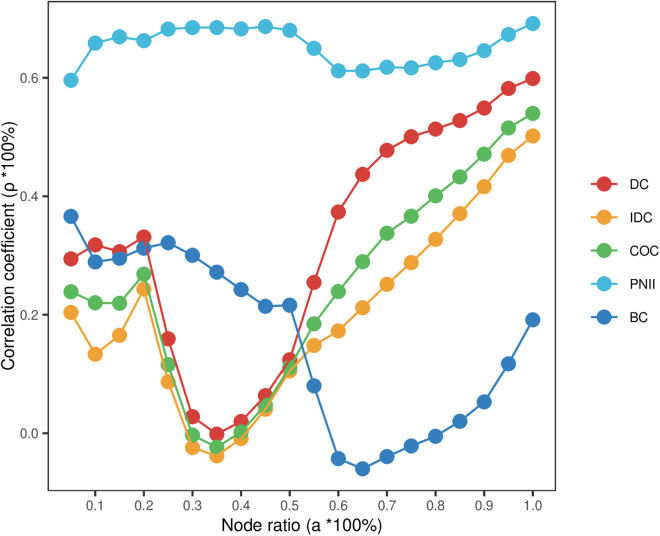
Evaluation effect of each network under different node ratios.

We further investigated the correlation between patent nodes with different ranking percentages, as determined by each network’s PNII, and the actual propagation influence on ranking outcomes. Like **Fig 4**, the PNII results of different networks are ranked from highest to lowest score and then correlated with nodes with a node ratio of $a=a_{0}\pm 0.05$. **Fig 5** shows that, in general, CPC evaluation results are relatively consistent under different node proportions. The evaluation results of CPC can make better use of the information provided by different relationships, so as to optimize the evaluation results of patent relational value.

As the ratio of nodes changes, the correlation between DC, IDC, and COC and the actual impact results shows a similar trend. BC had the worst correlation. This is because DC, IDC, and COC come from forward citing relationship, while BC comes from backward citing relationship. The backward citation relationship is opposite to the direction of propagation. When the node ratio of BC is 0.1, the correlation is larger, reflecting that a few high-value patents occupy the hub of the network, and have the characteristics of high indegree and outdegree.

### 4.5. Evaluation effect of different indicators

Patents have both relational value and individual value, depending on the perspective from which we view patent information. To explore the difference between patent relational value and individual value, we compare the impact of PNII and patent attribute index on patent value evaluation. We selected three indicators, the number of IPC classification numbers (IPCs), the number of patents in the same family (Family) and the number of patent holders (Holders). The number of IPC classification number describes the technical value attribute of a patent, which reflects the positive correlation between the technical coverage and the value of patent. The number of patents in the same family describes the attribute of economic value of patents. This indicator means the number of applications for the same invention in different countries, and the more patents in the same family, the higher the quality of the patent. The number of patent holders describes the value attribute of a patent. Generally, the more the patent holders, the more attention is paid to the value of the patent.

**Table 7** gives the descriptive statistics of the scores of different indicators. Compared with PNII, the three indicators cannot well reflect the “long tail distribution” of patent value. This is because the patent index score is generally discrete data, and the difference between the index scores of different patents is not obvious. The importance obtained by the patent network is generally continuous data, reflecting the scale-free characteristics of the patent network.

**Table 7:** Descriptive statistics of different indicators.

Index	IPCs	Family	Holders	PNII	$\beta_{0}$
Min.	1.000	1.000	1.000	0.000	1.000
1st Qu.	3.000	2.000	2.000	42.140	1.370
Median	7.000	6.000	4.000	295.360	2.250
Mean	7.263	8.229	4.507	1502.320	3.519
3rd Qu.	10.000	12.000	6.000	1119.710	4.060
Max.	57.000	63.000	22.000	90511.680	47.770
skewness	1.559	2.334	1.578	8.788	3.650
kurtosis	6.386	10.230	3.390	115.031	20.139

**Table 8** shows the correlation between different indicators and actual propagation influence. It can be found that the correlation between patent attribute indicators is high, and PNII based on relational characteristics is almost irrelevant to them. This may indicate that patent individual value and relational value have different perspectives from which to observe patent information, and they belong to different dimensions.

**Table 8:** Spearman correlation coefficients of different indicators.

Indicator	IPCs	Family	Holders	PNII	$\beta_{0}$
IPCs	1.000	0.843	0.647	−0.082	−0.125
Family		1.000	0.731	−0.075	−0.109
Holders			1.000	−0.026	−0.071
PNII				1.000	0.691
$\beta_{0}$					1.000

**Fig 6:**
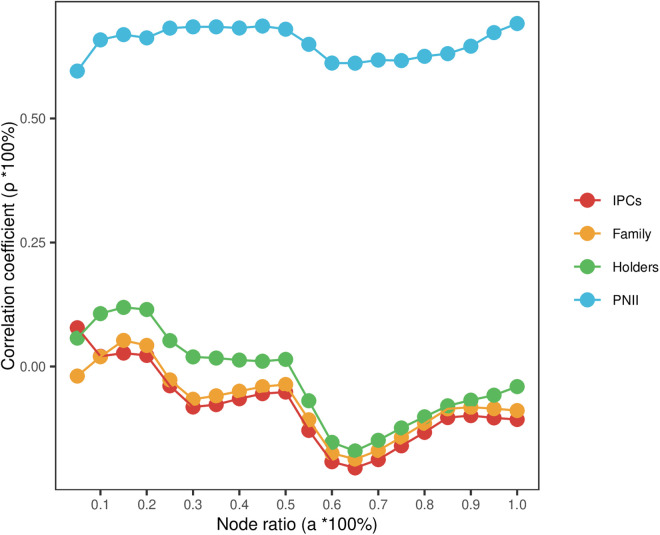
Evaluation effect of each indicator under different node ratios.

We further investigated the correlation between patents with different ranking percentages, as determined by the evaluation results of different metrics, and their actual impact on ranking results. Like **Fig 4**, the evaluation results of different indicators were ranked from highest to lowest, and then correlated with nodes whose node ratio is $a=a_{0}\pm 0.05$. As can be seen from **Fig 6**, the attribute indicators of patents are not correlated with the actual influence of patents in the network. This finding is consistent with the conclusion in **Table 8**, that the attribute indicators and citation relationships of patents measure different dimensions of patent information.

## 5. Conclusions and limitations

We constructed a comprehensive patent citation (CPC) network and employed the quantification of the degree of similarity between patents and their neighboring node pairs to delineate the importance of each node’s local network structure. Building on this approach, we introduced the PNII as a tool for evaluating the relational value of patents within specific technological domains. This method represents an advancement in patent relational value assessment from the viewpoint of network science.

The findings validate that the patent relational value evaluation method, grounded in the significance of patent network nodes, yields precise outcomes. This study suggests that the importance of a patent node should be assessed not only by its degree but also by the degree of similarity among the patent pairs that surround it, forming its neighborhood. The configuration of neighboring nodes mirrors the citation relationships between technologies, shedding light on the evolutionary dynamics of patent networks and effectively characterizing node importance. Both theoretical derivation and empirical analysis demonstrate that a method integrating node degree with the similarity of neighboring node pairs can effectively capture the value of patent relationships and the impact of network structure, leading to accurate evaluation of patent relational value.

Additionally, the approach leveraging the SIR model can be applied to assess the outcomes of patent relational value evaluations. The analysis examines the evaluation efficacy of various indicators across different propagation probabilities and node ratios. It reveals that the PNII yields more precise and consistent evaluation results than other centralities, thereby addressing some of the challenges in patent evaluation.

There are still limitations and shortcomings in this study. On the one hand, in the process of processing the networks, all types of citation relationships were considered as binary networks for merging, which may also affect the structural functions of different network types and may also cause errors in the evaluation results of patent value. On the other hand, this study only compared the evaluation results of some common network centralities, and did not use other indicators for further validation.

## Supporting information

S1 File (XLSX)
